# Advantages of continuous genotype values over genotype classes for GWAS in higher polyploids: a comparative study in hexaploid chrysanthemum

**DOI:** 10.1186/s12864-016-2926-5

**Published:** 2016-08-24

**Authors:** Fabian Grandke, Priyanka Singh, Henri C. M. Heuven, Jorn R. de Haan, Dirk Metzler

**Affiliations:** 1 Genetwister Technologies B.V., Wageningen, The Netherlands; 2 Fakultät für Biologie, University of Munich (LMU), Munich, Germany; 3 Institute for Molecules and Materials (IMM), Radboud University, Nijmegen, The Netherlands; 4 Animal Breeding and Genetics, Wageningen UR, Wageningen, The Netherlands

**Keywords:** Association study, Polyploids, Linear regression, Bayz, Partial least squares, Continuous genotypes

## Abstract

**Background:**

Association studies are an essential part of modern plant breeding, but are limited for polyploid crops. The increased number of possible genotype classes complicates the differentiation between them. Available methods are limited with respect to the ploidy level or data producing technologies. While genotype classification is an established noise reduction step in diploids, it gains complexity with increasing ploidy levels. Eventually, the errors produced by misclassifications exceed the benefits of genotype classes. Alternatively, continuous genotype values can be used for association analysis in higher polyploids. We associated continuous genotypes to three different traits and compared the results to the output of the genotype caller SuperMASSA. Linear, Bayesian and partial least squares regression were applied, to determine if the use of continuous genotypes is limited to a specific method. A disease, a flowering and a growth trait with *h*^2^ of 0.51, 0.78 and 0.91 were associated with a hexaploid chrysanthemum genotypes. The data set consisted of 55,825 probes and 228 samples.

**Results:**

We were able to detect associating probes using continuous genotypes for multiple traits, using different regression methods. The identified probe sets were overlapping, but not identical between the methods. Baysian regression was the most restrictive method, resulting in ten probes for one trait and none for the others. Linear and partial least squares regression led to numerous associating probes. Association based on genotype classes resulted in similar values, but missed several significant probes. A simulation study was used to successfully validate the number of associating markers.

**Conclusions:**

Association of various phenotypic traits with continuous genotypes is successful with both uni- and multivariate regression methods. Genotype calling does not improve the association and shows no advantages in this study. Instead, use of continuous genotypes simplifies the analysis, saves computational time and results more potential markers.

**Electronic supplementary material:**

The online version of this article (doi:10.1186/s12864-016-2926-5) contains supplementary material, which is available to authorized users.

## Background

Many agriculturally and horticulturally important crops are polyploid [1, 2]. Polyploids have multiple sets of chromosomes and have arisen by extensive genomic alteration and genome duplication [3, 4]. Diploidization, the differentiation of duplicated loci, converts most polyploids back to diploids on the long term [5, 6]. The phenomenon of polyploidy results in complex genomic architecture in many flowering plants thus complicates genomics-based breeding [7]. Research in polyploids is also limited by the available methods and technologies [8]. Most bioinformatic tools have been developed for diploids and cannot be applied to higher ploidy levels. Recently, several methods have been developed to overcome this limitation, but most of them are restricted to tetraploids [9, 10]. Association studies aim to determine a genetic origin for a phenotypic trait [11]. While phenotyping is independent of the ploidy level, genotyping has been identified as a bottleneck in breeding of polyploid crops [12]. Genotyping describes the process of determining an organism’s genotype [13] and is known to be erroneous [14]. It involves the extraction of genetic material, molecular biological processes and the assignment of genotypic classes.

**Fig. 1:**
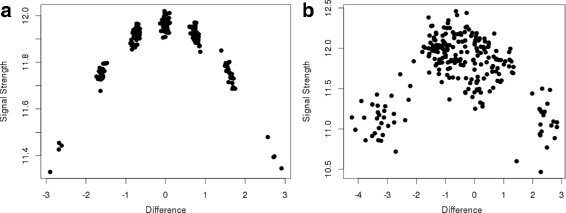
Example probe. Example of genotype values for a hexaploid probe and 228 samples. The x-axis shows the difference between the signals of the two alleles. The y-axis shows the average signal strength per sample. The left and right sides show simulated and real data, respectively. **a** The simulation demonstrates how the seven genotype classes cluster into groups. **b** The real data shows the full segregation over the whole spectrum, but no clustering into seven genotype classes

The latter one is also referred to as genotype calling and is a challenging task for polyploids. While there are many methods available for diploids [15], only three open access tools have been developed for polyploids, namely fitTetra, beadarrayMSV and SuperMASSA [12, 16, 17]. The former two are restricted to tetraploids and optimized for data sets originating from Illumina GoldenGate™ and Illumina BeadArray™, respectively. Subsequently, they underperform for data originating from other technologies [16]. SuperMASSA is a web tool that requires upload of individual data files for each SNP resulting in a poor performance. Further, the source code is not available and the algorithm cannot be validated. Density-based spatial clustering algorithms like OPTICS and DBSCAN [18, 19], were successfully applied in hexaploid wheat [20]. Preliminary analysis showed that they did not succeed for our genotypes, because the data points do not segregate into clusters, which can be distinguished based on density (Fig. 1).

Generally, determination of genotype classes (genotype calling) reduces noise and leads to better associations. In polyploids, this task is not as straightforward and the advantage of noise reduction is reversed by the high risk of misclassification, i.e. assignment of wrong genotypes to samples. Therefore, we skipped genotype calling and used the continuous genotypic values (compare Eq. 6) directly. The aim of this study was to use these continuous values to detect probes associating to three traits in hexaploid chrysanthemum and compare the results to genotype classes to evaluate the advantage of our approach. The traits have been selected to represent distinctive types (disease resistance, flowering and growth) and heritabilities (0.51, 0.78 and 0.92). We applied linear regression (LR), bayz (Bayesian regression) [21, 22] partial least squares regression (PLSR) [23, 24] and compared the results to avoid methodological bias. We showed that the assignment of genotype classes would not improve our findings, but lead to misclassification. In this article we demonstrate that we are able to use continuous genotype values to detect associations with three different traits in hexaploid chrysanthemum.

## Results and discussion

**Fig. 2:**
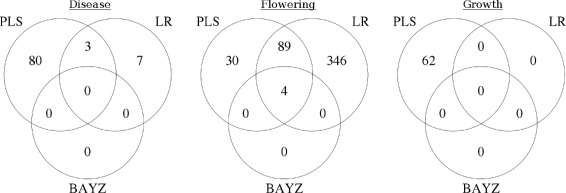
Result comparison. Venn diagrams of significant probes for the disease (**a**), flowering (**b**) and growth (**c**) trait. The significance thresholds for LR(*q*-value), bayz (BF) and PLS(VIM score) were ≤0.01, ≥10 and ≥2, respectively

We applied LR, bayz and PLSR to identify significant probes associated with the disease, flower and growth traits (Additional file 1). Later, we compared sets of significant probes identified by the above mentioned methods. Figure 2 gives an overview about total numbers of associated probes per method and overlap between them. We repeated the LR analysis with genotype calls by SuperMASSA and compared the results. Further, we simulated datasets with the same properties of our real dataset to determine the expected number of significant markers.

### Disease trait

**Fig. 3:**
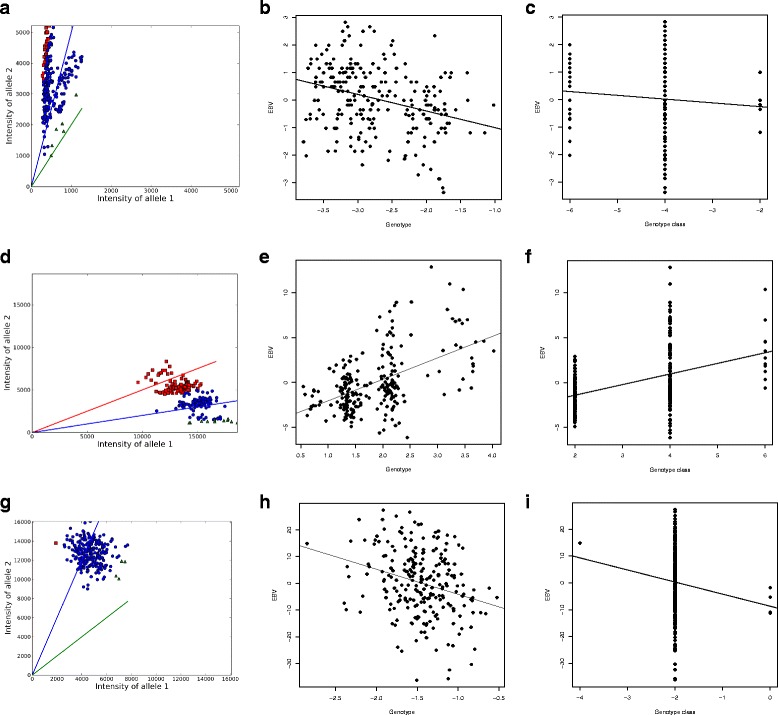
Comparison of genotype calls and continuous genotypes. Comparison of continuous values and genotype calls for three selected probes. **a**-**c**, **d**-**f** and **g**-**i** are the disease, flowering and growth trait, respectively. **a**, **d** and **g** were generated by SuperMASSA, based on the signal intensity values of the two alleles. The shapes and colors represent genotype clusters. The expected cluster centers are indicated by solid lines. **b**, **e** and **h** show the correlation of the raw genotype values with the corresponding EBVs. The solid lines represent the LR. **c**, **f** and **i** show the correlation of the genotype classes with the EBVs. The solid lines represent the LR

LR detected ten significant probes (*q*-value ≤0.01) when we used the continuous values. We called the genotype classes with SuperMASSA and repeated the LR analysis leading to 2 significant probes. We compared the results to see which approach worked better. An example is shown in Fig. 3(a-c). The axes in A represent the raw values of the two alleles. Three genotypes (red squares, blue circles and green triangles) were identified and assigned to the samples. The three lines represent the expected angles for each cluster center. The clusters identified by SuperMASSA (colors) do not match the groups that are indicated by the shape of the scatter plot. We consider the genotype calling as failed in that case. The blue cluster ranges over two groups, while the green cluster consists of outliers of the blue cluster. LR of the genotype class values with the phenotypes results in a p-value of 0.222 (Fig. 3c). Hence, the probe would be classified as non-significant, although it has not been corrected for multiple testing. The p- and *q*-values of the LR of the continuous genotypes for the same probe were 9.97×10^−7^ and 0.0078, respectively (Fig. 3b).

Additional file 2 shows a comparison of the significance values by SuperMASSA and the continuous values. Taken together, genotype calling distorted genotypic INFORMATION for some markers and the prevented their correctassociation. The desired noise reduction, which improves associations, could not be achieved. PLSR detected 83 probes with variance importance (VIM) scores ≥2. Three of them overlap with the LR results. bayz did not find any significant probes with a Bayes factor (BF) threshold over 10. Even with a less-strict threshold of 5 there were no findings (compare Additional file 3).

### Flowering trait

LR detected 439 significant probes for the flowering trait with the continuous values. SuperMASSA genotype calls led to 332 significant probes (Additional file 4). Again, the continuous genotypes generally lead to lower *q*-values than the genotype calls (compare Additional file 2). We simulated the experiment with varying numbers of significant probes (2-10) 100 times each. That way, we could observe how many significant probes we would detect if the true genotypes are known. The simulation results are shown in Additional file 5 and show that we expect around 100-2000 significant probes. Hence, our association results are in the correct magnitude. For our dataset continuous genotype values are advantageous over genotype classes because we obtain more significant probes and are less likely to miss trait related probes. However, our method is not too insensitive and does not result in thousands of false positive markers. In fact, the simulation study detected even more false positives. It reduces the number of potential candidate probes from 55,825 to 439.

An example probe for the flowering trait is shown in Fig. 3d-f. SuperMASSA identified three different genotype classes. Continuous genotype values indicate four genotype classes, roughly centered at 0.75, 1.5, 2.2 and 3.5 (Fig. 3e). In contrast, the genotype classes by SuperMASSA combine the first two clusters (Fig. 3d). The blue one contains samples of the first and third cluster, which leads to its spread over the whole DEBV range (Fig. 3f). This leads to a lower p-value, but the probe is still highly significant. Nevertheless, in other cases this difference might determine whether the null hypothesis can be rejected or not.

**Fig. 4:**
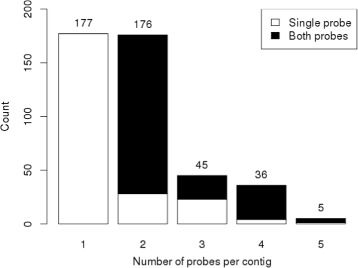
Overview of contigs and probes for the flowering trait. Probe and contig distribution of the significant markers of the flowering trait. The five bars represent the number of markers that lay in the same contig. The colors distinguish between markers where both or only one of the probes were significant and are more reliable. Multiple markers from the same contig indicate its association to the trait

Figure 4 shows the detailed distribution of both probes and contigs for the 439 probes. 206 probes were duplicates, i.e. one codes for the forward and one for the reverse strand of 103 SNPs. The significance of both probes adds to the probability of the SNPs association. Accordingly, the 233 remaining probes code for unique SNPs. There are multiple scenarios when only one probe is selected. First, there is an additional SNP within the primer sequence of the failing probe and the hybridization is disturbed. Secondly, the other probe had a similar signal, but was above the significance threshold and filtered out. Thirdly,the SNP is not associated to the trait and the probe was selected erroneously. Lastly, the other probe has been filtered out in a preprocessing step, based on the segregation range of the *θ* values.

The probes were selected so that not more than three SNPs (six probes) lay on one contig. Thus, it is unlikely to detect two or more significant SNPs from the same contig by chance. The fact that we found up to three SNPs from the same contig is a good indicator for real association. We detected 5 probes from one contig, coding for three different SNPs. The white area in the second bar in Fig. 4 shows 28 single probes, which code for different markers, but are located on the same contig. Accordingly, the missing second probe is not required to proof association.

The PLSR and bayz associations detected 123 and 4 significant probes, respectively. The four probes, detected by bayz overlap with the LR and PLSR results. In addition there is an overlap of 89 probes between PLSR and LR. However, the associating probes have low *R*^2^ values and explain only parts of the phenotypic variance. Table 1 shows the four probes, which have been identified by all three methods. The scores do not correlate because the methods base on different approaches. The *R*^2^ values are obtained from the LR association.

**Table 1:** Overlapping significant markers between all three methods for the flowering trait

Marker	*R* ^2^	*q*-value	BF	VIM
AX-89300609	0.31	1.72×10^−15^	11.03	3.23
AX-89215144	0.26	8.90×10^−13^	15.49	2.90
AX-89213862	0.18	1.74×10^−8^	24.82	4.12
AX-89256548	0.09	5.16×10^−6^	12.27	3.45

### Growth trait

LR did not result in any significant probes for the growth trait. Growth is known to be a polygenic trait [25]. Hence, we did not expect single probes to show strong association. The association with the SuperMASSA genotype calls did not output any significant probes, either (Additional file 6). An example probe is shown in Fig. 3g. All but five samples were assigned the same genotype class. We expect monomorphic probes, but in Fig. 3h we see that the genotype values span a large portion of the negative *θ* range. Thus, we expect multiple genotype classes within that cloud of data points. However, they could neither be determined manually nor computationally. bayz did not detect any significant probes, either. In contrast, the PLSR association detected 62 significant probes. It includes low effect markers as well and is not limited to single loci. For polygenic traits PLSR is therefore advantageous.

### General

We could have applied sparse partial least squares (SPLS) regression to deal with very high-dimensional data. The expected number of associating probes would be even higher than. We decided against it because the number of false positives and low impact probes (based on explained variance from the LR analysis) would have increased significantly. PLSR is less restrictive in the growing and disease trait but not in the flowering trait.

EBVs are established metrics in breeding, but have been criticized for genome-wide association studies (GWAS) because their naïve usage reduces power, increases the false positive rate and misestimates effect sized of quantitative trait loci (QTL) [26]. Hence, we used deregressed EBVs to account for fixed effects and repeated measurements, as described by Garrick et al. [27].

SuperMASSA misclassifies genotypes in some cases because it assumes clusters of equal distance at fixed positions. Instead, the data does not necessarily segregate into clusters (Fig. 3g). If clusters can be identified, they are not always at fixed positions, as assumed by SuperMASSA (Fig. 3d). Further, it does not account for outliers and assigns genotype classes to every sample. This results in clusters of only one sample in some cases and does not represent the genotype. Taken together, SuperMASSA does not cluster the data points properly for all probes. It seems to be optimized for data produced with two technologies and therefore performs less well on other data sets. The same situation was described by Voorips et al. [16], when they compared fitTetra and beadarrayMSV. Thus, genotype calling has no advantage for polyploid data generated with the Affymetrix Axiom™ technology, as long as no method works properly. This underpins our preliminary analyis, which showed that the resolution of the signal intensities is not large enough to distiguish between the increased number of genotype classes expected in hexaploids (Fig. 1). To this end, association of the continuous genotypes is currently the best method.

Genotyping by sequencing (GBS) results in similar datasets, where the continuous genotypes are replaced by read counts. In general, our approach can be applied to this kind of data, but it is limited to bi-allelic SNPs and requires an extension to account for multi-allelic SNPs. Assuming an additive genetic effect [28] and a multi-allelic SNP, *θ* needs to be upgraded to *θ*_*i*_:

1 $$ \theta_{i} = \log_{2}(G_{i}) - \sum\limits_{j=1, j\ne i}^{4} \log_{2}(G_{j})  $$

where *G* is the set of alleles and *G*_*i*_ is the read count of allele *i*. Alternatives to the log-transformations might be more effective and need to be investigated [17].

The higher the ploidy level, the smaller is the advantage of genotype classes over the continuous values. With increasing numbers of genotype classes, the effect of noise reduction declines. For instance, in a diploid we expect the clusters AA, AB and BB at around 2, 0 and −2, respectively. A value of 1.2 would be assigned to cluster AA, so we correct for a large proportion of the signal. For any ploidy level *n* we expect up to 2*n*+1 clusters on a similar range, because the overall signal strength is limited by the used technology (e.g. amount of genetic material, GBS read depth). Consequently the distances between clusters decrease and the correction accounts for smaller proportions of the signal. In addition, the risk of misclassifications increases, because there is less tolerance for variation in the signal intensity or clusters overlap. Further, the distribution of genotype values approximates a continuous distribution with increasing ploidy levels. Figure 1 shows an example of a simulated hexaploid marker and one from the real data set. We were not able to identify the genotype classes for all samples in that case, because the clusters are indistinguishable. Nevertheless, the data points spread over the whole range of *θ* and provide genotypic information. From a biological perspective, continuous genotypes are difficult to interpret, because the number of alleles is discrete and should fall in one of the genotype classes. One explanation are tri- and tetra-allelic SNPs, where more than two nucleotides are present at the same position [29, 30]. If they are measured with bi-allelic technology (e.g. genotyping arrays), the sum of the two allele counts does not necessarily add up to the expected number (ploidy level). Alternatively, we might observe fractionation, the deletions in sub-genomes of allopolyploids [31]. Both result in data points outside of the expected clusters. For the association we mean-centered the genotype values.

Skipping genotype calling leads to further challenges with current linkage mapping and haplotype phasing methods, because they require genotype classes. Nevertheless, the choice of tools that work for polyploids is very limited anyways and new solutions need to be developed. Further, low-coverage sequencing and imputations of genotypes add more difficulties [32].

## Conclusions

We showed that continuous genotype data can be used successfully in an association study of a polyploid crop and validated our findings in a simulation study. Application of different regression tools show that our approach is not limited to a specific method, but the results vary to a large extend.

Genotype calling leads to misclassification and false association results in some cases, where significant markers could not be detected. However, the majority of markers lead to similar results with genotype classes and continuous values, indicating that genotype calling is not adversely in general. In this study genotype calling has no advantage and can be skipped unless better methods are developed. Instead, use of continuous genotypes simplifies the analysis, saves computational time and results more potential markers. Nevertheless, the overlapping clusters of the given data set remain a challenge and the use of continuous genotypes is a successful solution to that problem.

## Methods

A hexaploid chrysanthemum population consisting of 228 F1 offspring was used for our study. The cultivated plant material was provided by Dümmen Orange and all experiments have been performed according to legal guidelines.

### Phenotypes

Three different traits have been used, as shown in Table 2. They represent the three areas that are relevant in a horticultural crop association study: disease, growth and flowering. Further, they span a wide range of heritability values. Details about the traits are not provided, because they are confidential and not important for the methodology itself. All traits’ distributions are bell shaped and can be approximated by a normal distribution. The replicated measurements have been transformed into deregressed estimated breeding values (DEBV).

**Table 2:** Overview of traits (DEBV)

Trait	*h* ^2^	Mean	SD	Range	Samples
Disease	0.51	0.05	1.2	6.19	228
Flowering	0.78	0.00	3.3	19.01	228
Growth	0.92	0.21	12.2	63.57	228

The estimation of the breeding values (EBVs) was performed using ASReml-R [33]. The EBVs for the individuals were derived by fitting a mixed linear model using the REML (residual maximum likelihood) procedure (Additional file 7). The asreml model was fitted to optimally use the information available for each individual, while simultaneously adjusting for environmental effect i.e. block and plate numbers. The mixed model for calculation of EBVs can be presented as

2 $$ y_{i} = \alpha + {\beta^{1}_{i}} + {\beta^{2}_{i}} + g_{i} + e_{i}  $$

Where *y*_*i*_ is the observed trait value, *α* is the population mean, ${\beta ^{1}_{i}}$ is the fixed block effect, ${\beta ^{2}_{i}}$ is the fixed plate number effect. *g*_*i*_ is the random accession effect, where $g \sim N(0,{\sigma _{g}^{2}})$ and *e*_*i*_ is the random error of the observed trait value, where $e \sim N(0,{\sigma _{e}^{2}})$. In order to calculate DEBV s as described by Garrick et al. [27], the predictive error variance (PEV) was calculated from the model parameters. Here, we have used variances of EBVs as a measure for PEV. The DEBV s were calculated using

3 $$ debv_{i} = \frac{ebv_{i}}{{r_{i}^{2}}}  $$

With

4 $$ {r_{i}^{2}} = 1 - \frac{PEV_{i}}{{\sigma_{g}^{2}}}  $$

and

5 $$ PEV_{i} = var(ebv_{i})  $$

where ${r_{i}^{2}}$ is the reliability of the EBV of plant *i* and ${\sigma _{g}^{2}}$ is the additive genetic variance.

### Genotypes

The genotypes were measured with a customized Affymetrix Axiom™ microarray. It provides ∼100*k* probes for hexaploid chrysanthemum. We filtered out probes with a *θ* range below 2, because association requires segregation. The final data set consists of 55,825 probes. Each SNP is represented by two probes, upstream and downstream, respectively. We genotyped 228 samples and preprocessed them with Affymetrix Power Tools [34]. This includes quantile normalization and transformation of the microarray measurements. The genotype calling step from Affymetrix Power Tools was not performed, because it is limited to diploids and cannot detect more than three clusters. The microarray provides one value for each of the two alleles for every probe. The two measurements *A* and *B* are transformed into difference values *θ*, where

6 $$ \theta = \log_{2}(B) - \log_{2}(A)  $$

and a signal strength *s* where

7 $$ s = \frac{\log_{2}(A) + \log_{2}(B)}{2}  $$

An example of a bi-allelic probe from a hexaploid chrysanthemum data set is shown in Fig. 1. The x-axis represents *θ*, the difference between the two alleles A and B. The values span the whole range of potential genotypes and represent seven different genotype classes. The leftmost samples are homozygous A, while the rightmost ones are homozygous B. The intermediates are heterozygous in varying proportions. The y-axis shows the mean signal strength s. The homozygous s values are lower, because logarithmic values are used.

The genotype calling was done with the web application of SuperMASSA without population-level information (http://statgen.esalq.usp.br/SuperMASSA/) [12]. The ploidy range was set from 2 to 6; the other parameters were used with the default parameters. We used the raw values of the two alleles as input. The resulting genotypes represented the numbers of the two alleles. For the association we used the difference between the counts of the first and second allele.

### Association methods

Three different methods to associate the continuous genotypic values with the phenotypes were used: LR, bayz and partial least squares regression (PLSR). The model to calculate the LR for all three traits was

8 $$ Y_{i} = \alpha + \beta x_{i} + \epsilon_{i}  $$

where *Y*_*i*_ are the DEBV s, *α* is the population mean, *β* is the regression coefficient, *x*_*i*_ the mean-centered, continuous genotype value and *ε*_*i*_ the residual error. The function lm from the R package stats (Version 3.1.3) with the default parameters was used for the regression [35]. The resulting p-values were transformed into *q*-values with the function *qvalue* of the R-package qvalue (Version 1.43.0) with default parameters [36, 37]. We applied a threshold of 0.01 to select the significantly associating probes.

The effect of each SNP was estimated using Bayesian Variable Selection method as implemented in the bayz software [21, 22] and described by Schurink et al. [38]. The applied method is similar to the *BayesCπ* method [39], except the prior of *π* was changed from a uniform(0,1) distribution to a slightly informative prior distribution ∼*Beta*(10,1). In bayz, shrinkage of allele effects was done by applying a mixture distribution. Many SNP effects were shrunk to nearly zero to obtain high sparsity in SNP effects and only a small part of the SNP effects were less severely shrunken, thereby identifying SNPs with important associations. The prior mixture distribution was

9 $$ a_{k}\left\{ \begin{array}{ll} N~(0,\sigma_{g0}^{2}), \text{with probability}~\pi_{0}\\ N(0,\sigma^{2}_{g1}), \text{with probability}~ \pi_{1}=(1-\pi_{0}) \end{array} \right.  $$

Where the ‘null’ distribution modeled the majority of SNP with (virtually) no effect using prior settings *π*_0_=0.99 and $\sigma _{g0}^{2}=0.001$. The second distribution modeled SNPs with large effects where prior settings were *π*_1_=0.01 and $\sigma ^{2}_{g1}=0.1$. Variances of the mixture distribution and other model effects were estimated using a uniform prior and sampled with a Monte Carlo Markov Chain (MCMC) using Gibbs sampling. The MCMC was run for 50,000 iterations with a burn-in of 10,000 iterations and a thin-interval of 200. A Bernoulli distribution specified probabilities for a SNP belonging to the ‘null’ or second distribution and proportions for the mixture were set to have a slightly informative prior distribution ∼*Beta*(10,1).

For the PLSR analysis we used the most significant probes of the LR analysis, based on F-statistic values ≥4. The numbers of probes were 4517, 4546 and 4957 for the disease, flowering and growth trait, respectively. We used the *pls* functions of the R package caret (version 6.0-47) [40]. The association was accomplished in three steps. First, the data was split into a calibration (80 %) and a test set (20 %). Second, the calibration set was used to select the optimal latent variables (LV). We repeated a 10-fold cross validation 20 times and assessed 1-10 LVs based on the lowest root mean squared error (RMSE):

10 $$ RMSE = \sqrt{\frac{1}{n} \sum\limits_{i=1}^{n} (\gamma_{i}-\hat{\gamma}_{i})^{2}}  $$

Where *n* is the number of samples, *γ*_*i*_ is the observed and $\hat {\gamma }_{i}$ is the predicted phenotypic value. In the third step the model was build and the significant probes were predicted based on their variable importance measurement (VIM) scores with a lower threshold of 2 [41, 42]. The scores were determined with the *varImp* function from the R-package caret [40]. The calculation is based on the weighted sums of the absolute regression coefficients.

### Simulation

We simulated 228 F1 offspring genotypes (55,825 probes on 18 chromosomes) based on the parental genotypes with PedigreeSim [43]. We selected 2 to 10 associating probes randomly and calculated phenotypic values *Y*_*i*_ for each offspring *i* with an adapted formular by Günter et al. [44]

11 $$ Y_{i} = \sqrt{1-\sum\limits_{j}\pi_{j}}*N(0,1) + \sum\limits_{j}a_{ij}\sqrt{\frac{\pi_{j}}{6*f_{j}(1-f_{j})}},  $$

where *π*_*j*_ is the explained variance $\left (\sum _{j}\pi _{j} \text { is the}\text {heritability}\right)$, *f*_*j*_ is the allele frequency and *a*_*ij*_ is the genotype of sample *i* for probe *j*. The heritability was set to 0.78 as for the flowering trait. Afterwards, we associated the simulated phenotypes with the genotypes using LR. This simulation procedure was repeated 100 times for each parameter combination.

## Abbreviations

BF, Bayes factor; PLSR, partial least squares regression; DEBV, deregressed estimated breeding value; GBS, genotyping by sequencing; LR, linear regression; MCMC, Monte Carlo Markov Chain; PEV, predictive error variance; REML, residual maximum likelihood; SNP, single nucleotide polymorphism; SPLS, sparse partial least squares

## Additional files

Additional file 1 Association scores. Tab-separated data file with *q*-values (LR), VIM-scores (PLS) and BFs (bayz) for all probes for all three traits. (TSV 4970 kb)

Additional file 2 SuperMASSA results. PDF including the plots of the significant calls of the LR analysis. (PDF 17 kb)

Additional file 3 Genotype calling Disease. PDF including 10 images of genotype calls produced with SuperMASSA. (PDF 735 kb)

Additional file 4 Genotype calling Flowering. PDF including 11 images of genotype calls produced with SuperMASSA. (PDF 773 kb)

Additional file 5 Simulation results. PDF including the simulation results. (PDF 33 kb)

Additional file 6 Genotype calling Growth. PDF including 12 images of genotype calls produced with SuperMASSA. (PDF 960 kb)

Additional file 7 Estimated Breeding Values. Tab-separated data file of the deregressed estimated breeding values for the Disease, Flowering and Growth trait for all 228 samples. (TSV 15 kb)
