# Supplementary figures and images for: Advantages of continuous genotype values over genotype classes for GWAS in higher polyploids: a comparative study in hexaploid chrysanthemum
Source: BMC Genomics. 2016 Aug 24;17(1):672. doi: 10.1186/s12864-016-2926-5 (PMC4995758; doi:10.1186/s12864-016-2926-5)

**Disease**

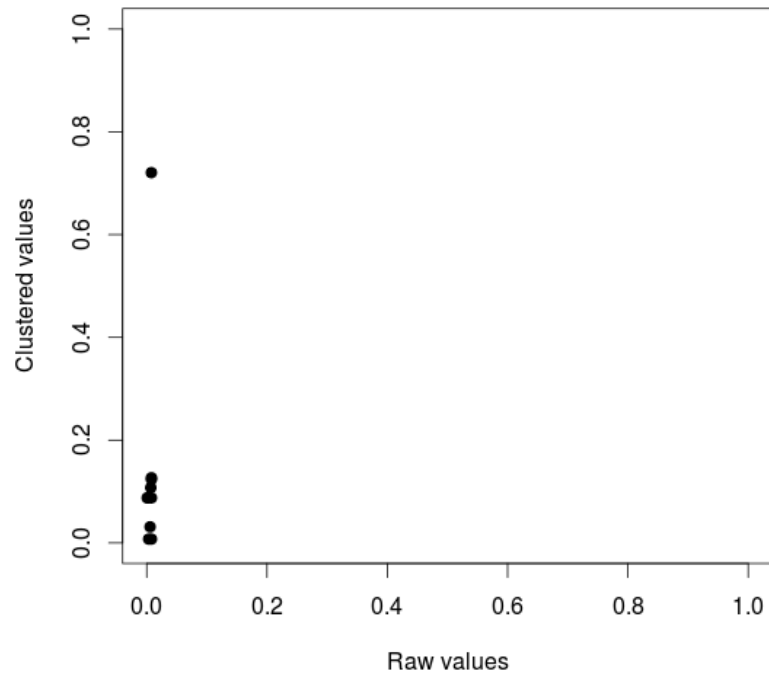

**Flowering**

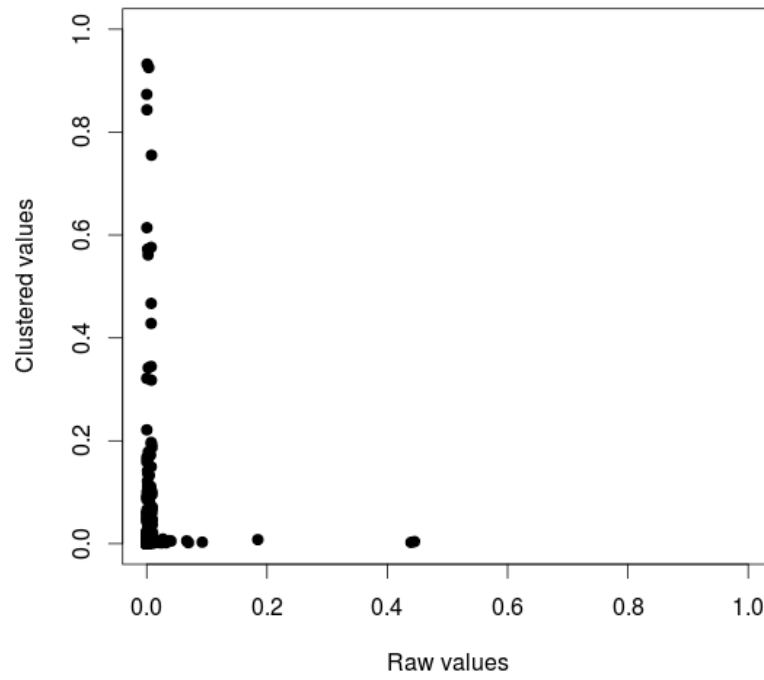

Supplement: Additional file 2 — SuperMASSA results. PDF including the plots of the significant calls of the LR analysis. (PDF 17 kb) [file 12864_2016_2926_MOESM2_ESM.pdf]

## SuperMASSA genotype calling for disease trait markers

- AX-89299922
- AX-89251911
- AX-89296762
- AX-89279384
- AX-89244354
- AX-89257336
- AX-89253330
- AX-89231276
- AX-89238198
- AX-89264014

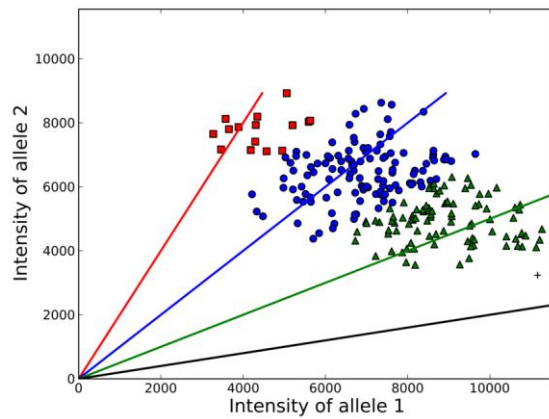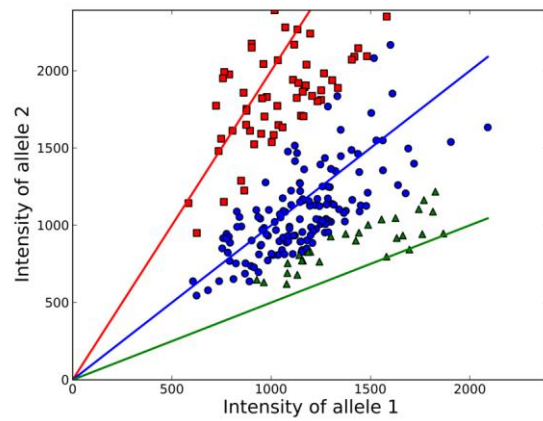

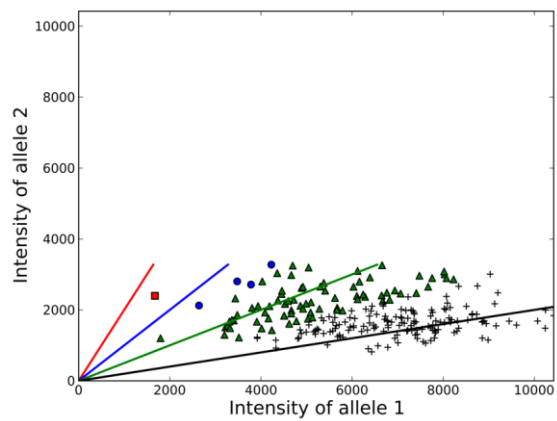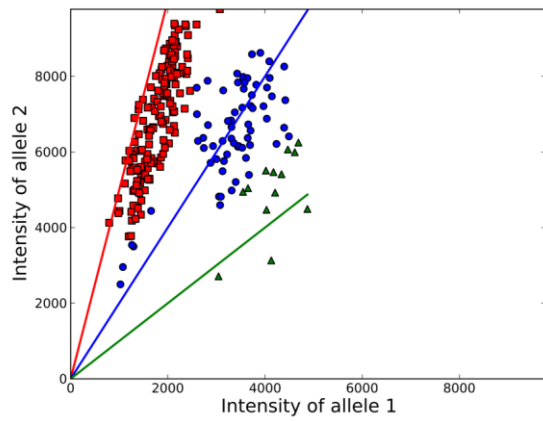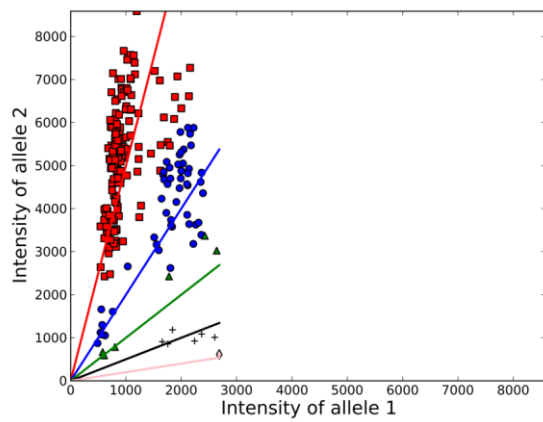

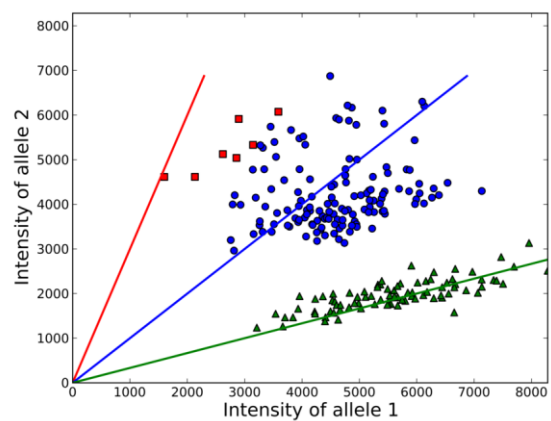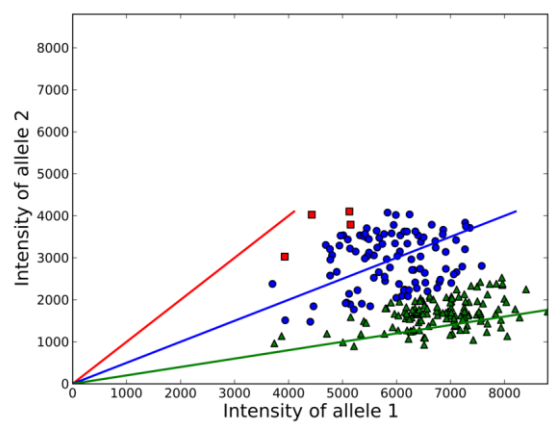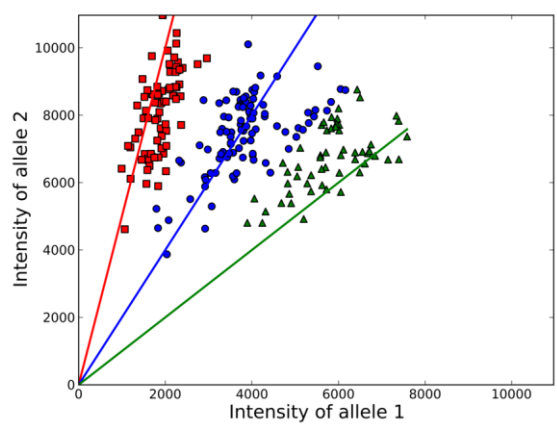

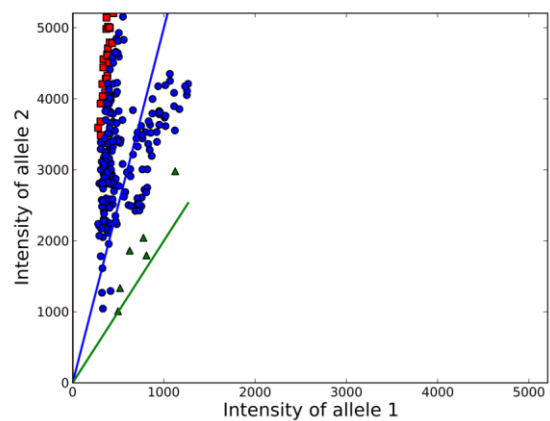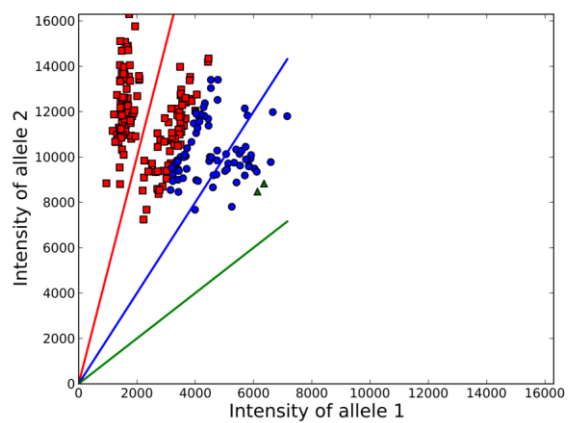

Supplement: Additional file 3 — Genotype calling Disease. PDF including 10 images of genotype calls produced with SuperMASSA. (PDF 735 kb) [file 12864_2016_2926_MOESM3_ESM.pdf]

### SuperMASSA genotype calling for flowering trait markers

- AX-89225990
- AX-89287826
- AX-89284793
- AX-89231611
- AX-89300609
- AX-89243551
- AX-89291422
- AX-89211288
- AX-89261288
- AX-89215144
- AX-89265144

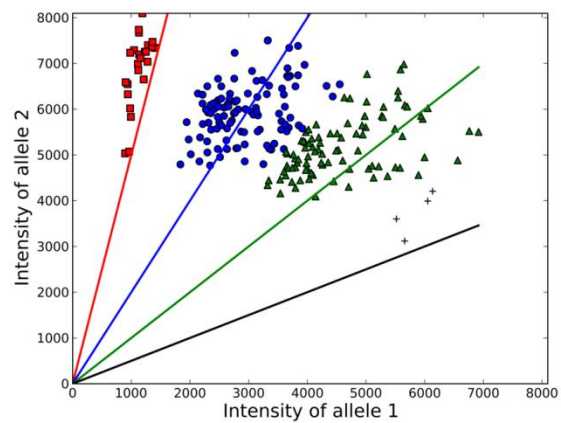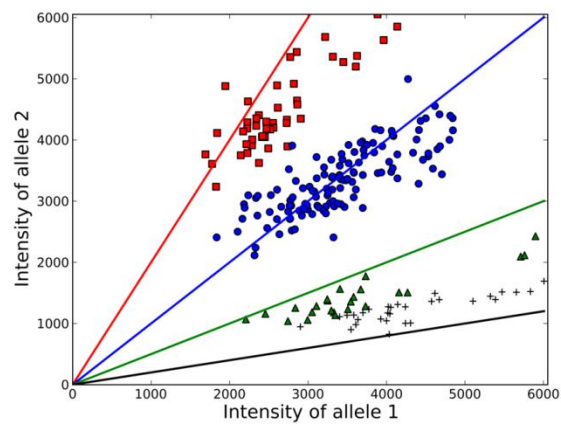

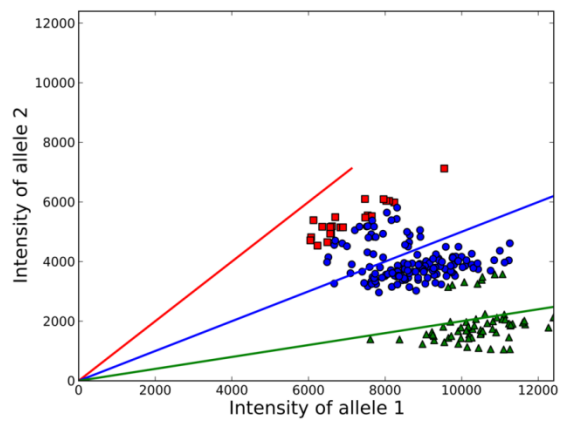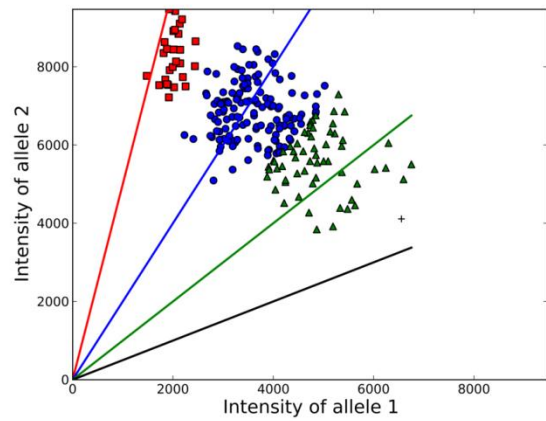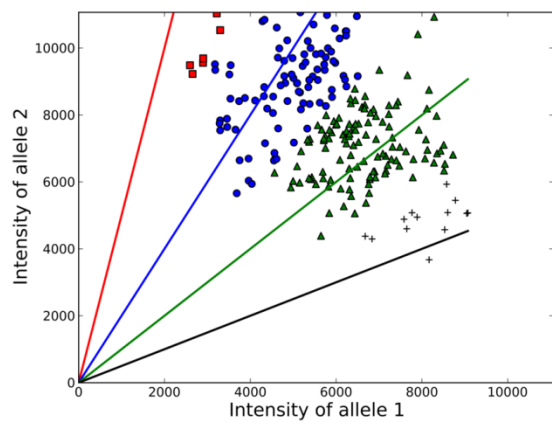

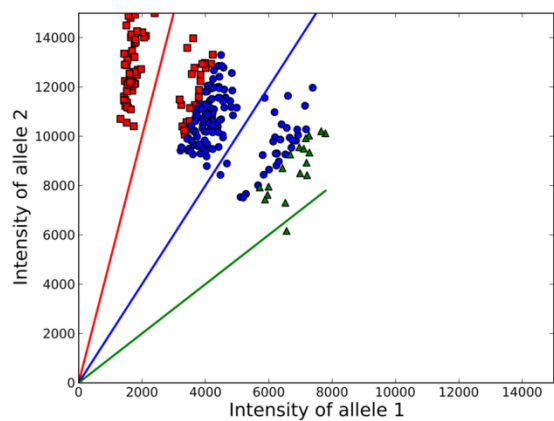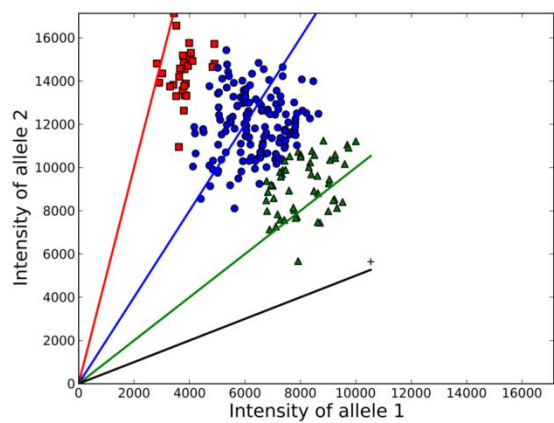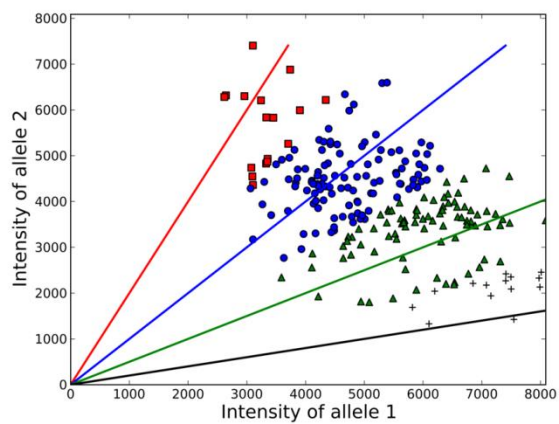

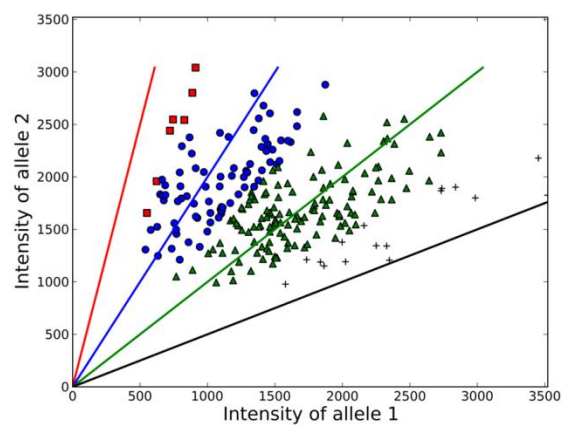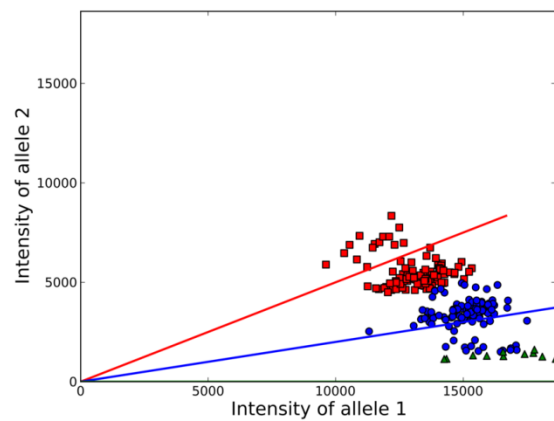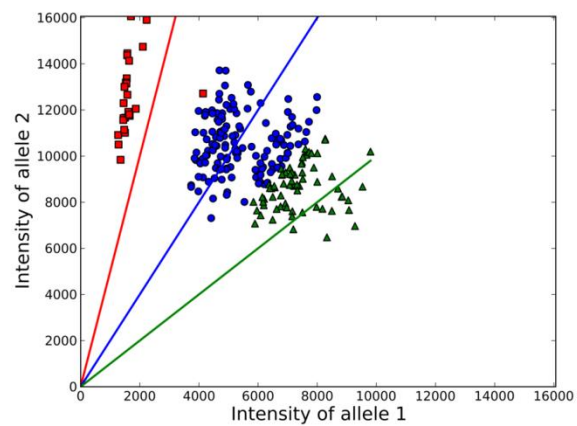

Supplement: Additional file 4 — Genotype calling Flowering. PDF including 11 images of genotype calls produced with SuperMASSA. (PDF 773 kb) [file 12864_2016_2926_MOESM4_ESM.pdf]

### SuperMASSA genotype calling for growth trait markers

- AX-89227964
- AX-89255868
- AX-89272774
- AX-89253659
- AX-89299519
- AX-89216469
- AX-89302464
- AX-89306355
- AX-89212314
- AX-89226287
- AX-89226270
- AX-89306258

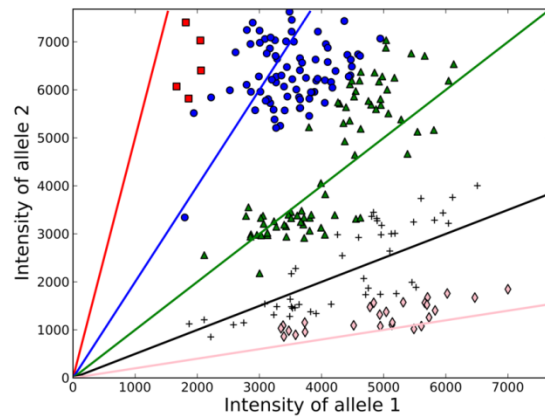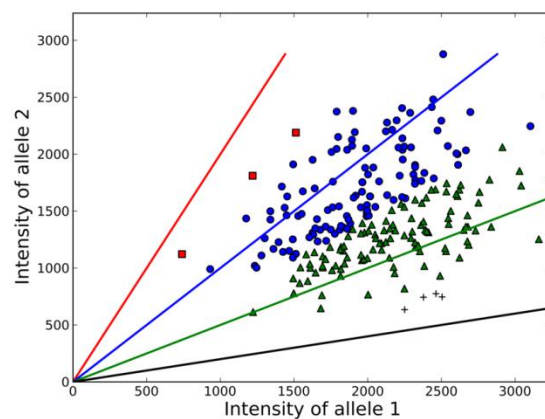

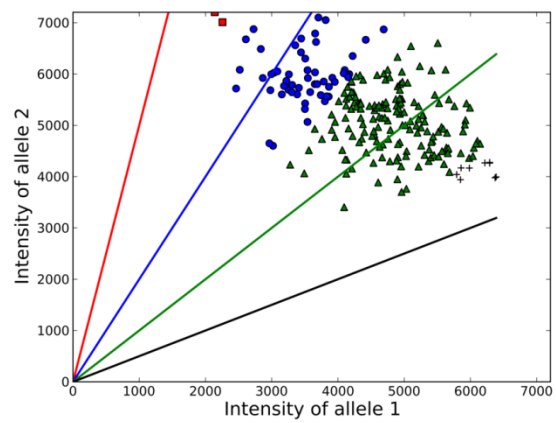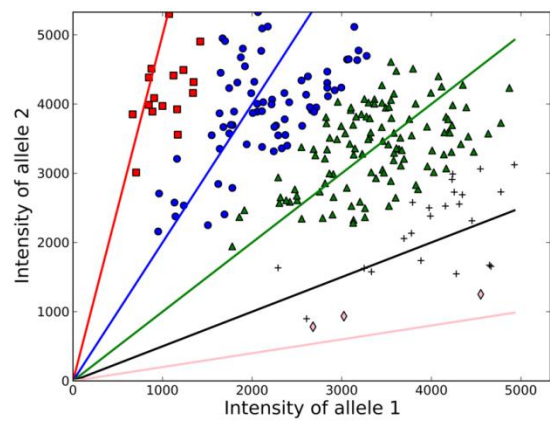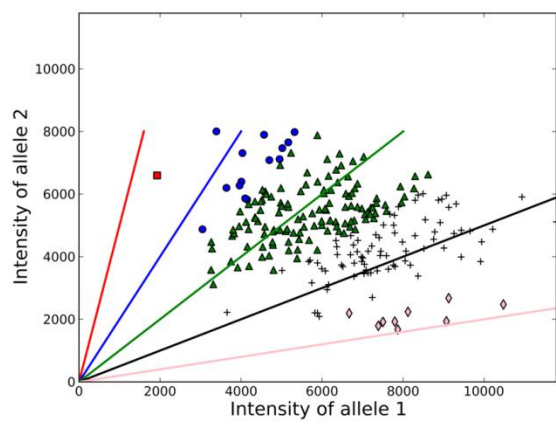

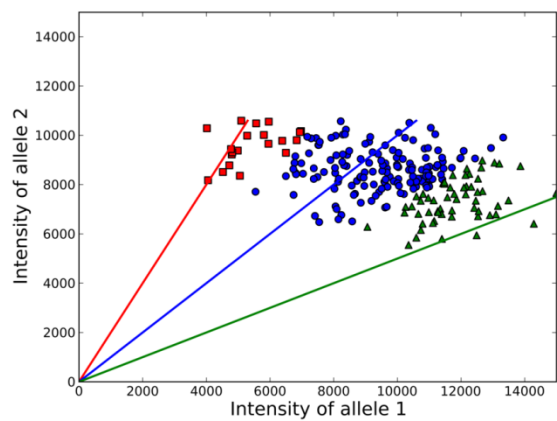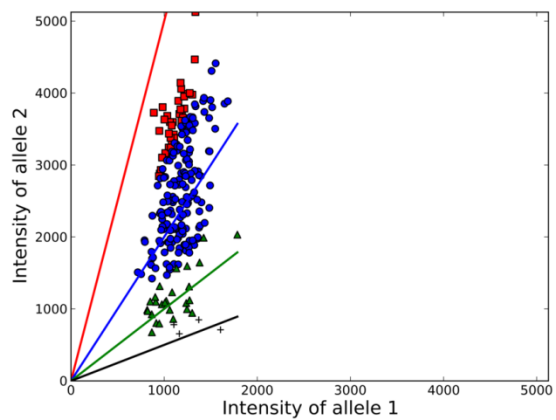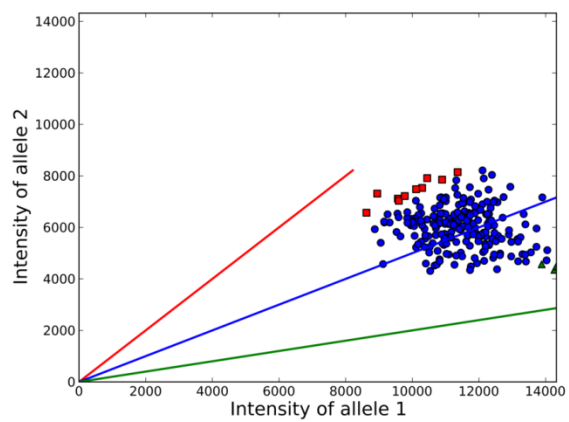

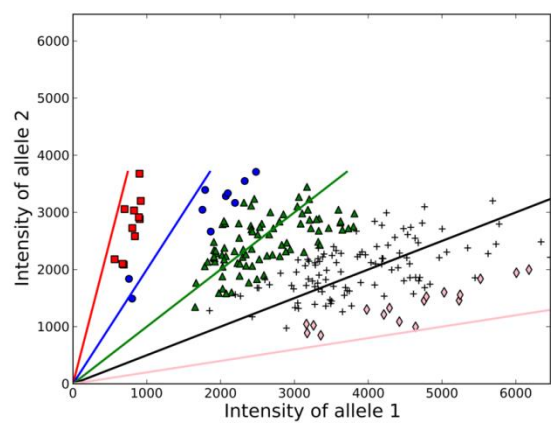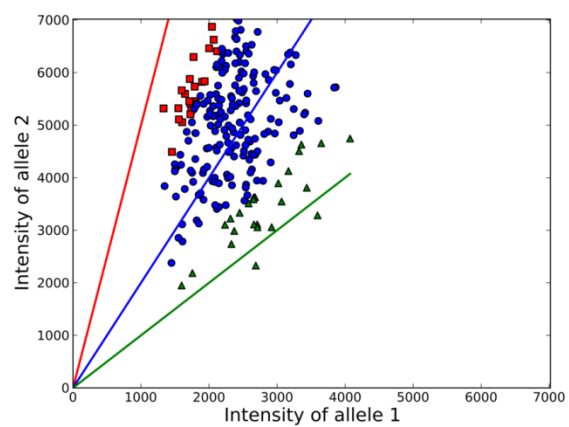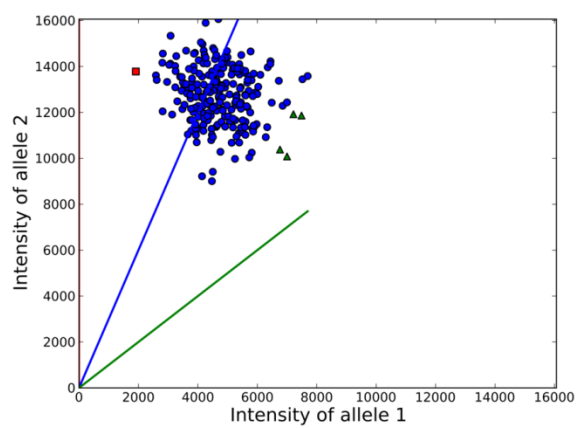

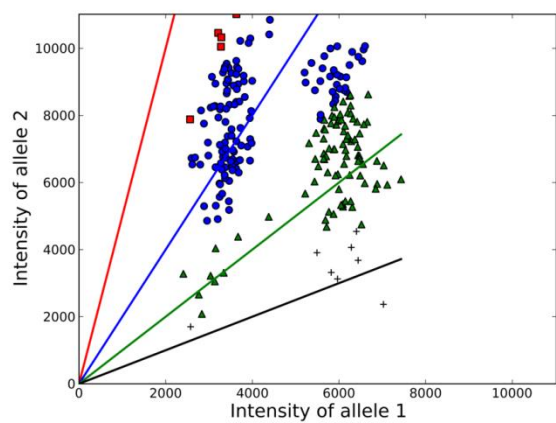

Supplement: Additional file 6 — Genotype calling Growth. PDF including 12 images of genotype calls produced with SuperMASSA. (PDF 960 kb) [file 12864_2016_2926_MOESM6_ESM.pdf]
