# Supplementary material for: Advantages of continuous genotype values over genotype classes for GWAS in higher polyploids: a comparative study in hexaploid chrysanthemum
Source: BMC Genomics. 2016 Aug 24;17(1):672. doi: 10.1186/s12864-016-2926-5 (PMC4995758; doi:10.1186/s12864-016-2926-5)
Supplement: Additional file 5 — Simulation results. PDF including the simulation results. (PDF 33 kb) [file 12864_2016_2926_MOESM5_ESM.pdf]

Simulation study results

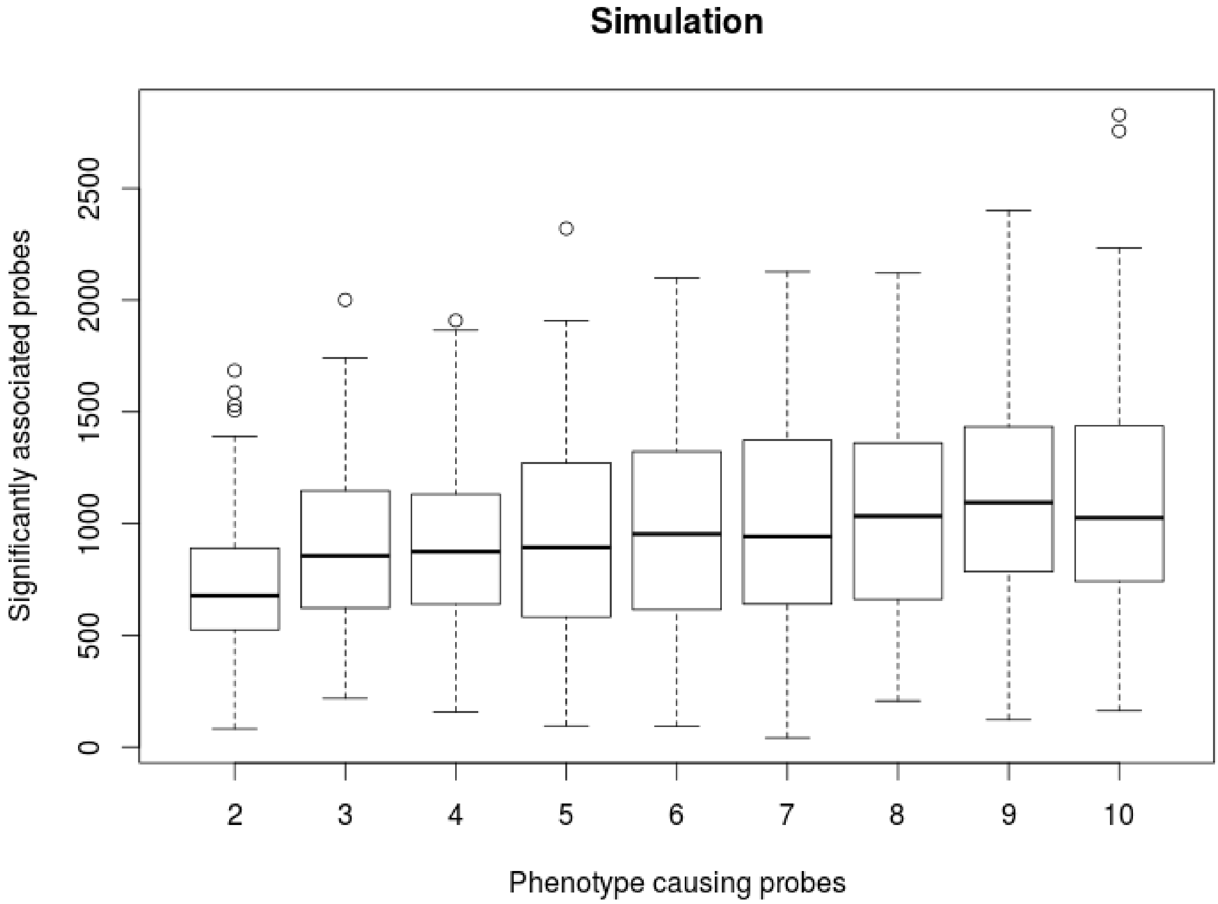

We simulated 9 datasets with 2 to 10 phenotype causing probes each. Each simulation was repeated 100 times. The boxplots show the distribution of significant probes per dataset. The circles and bold lines represent outliers and medians, respectively.
